# Sports venue digital twin technology from a spectator virtual visiting perspective

**DOI:** 10.3389/fspor.2023.1289140

**Published:** 2023-12-05

**Authors:** Ekaterina Glebova, Robert Book, Yiran Su, Marko Perić, Jonas Heller

**Affiliations:** ^1^ Université Paris-Saclay, CIAMS, 91405, Orsay, France; ^2^ Department of Sports, Faculty of Humanities, Sports and Educational Science, Physical Education and Outdoor Studies, Campus Bø, University of South-Eastern Norway, Bø, Norway; ^3^ Isenberg School of Management, University of UMass Amherst, Amherst, MA, United States; ^4^ Faculty of Tourism and Hospitality Management, University of Rijeka, Rijeka, Croatia; ^5^ Department of Marketing and Supply Chain Management, School of Business and Economics, Maastricht University, Maastricht, Netherlands

**Keywords:** digital twin, sports spectator, sports event, sustainability, virtual visiting, fan engagement, sports venue, digital avatar

## Abstract

The purpose of this article is to adopt a customer-centric perspective the and introduce digital twin technology as a solution for mega-sport event management. This conceptual model article focuses on the potentially drastic role of digital twin technology in modern sports events, explaining in detail different aspects of its impact. The main research question is “How (and why) do sports venue digital twin emerging technologies prospectively impact the sports spectators” customer experiences?” It contributes to understanding how and why sports venue digital twins make events more customer-centred by enhancing fan experiences and engagement. Subsequently, it aims to position digital twin technology as an innovative solution for mega-sport event management across various customer experience touchpoints. By examining the intersection of digital twins and sports events from a customer-centric lens, this article will elucidate the intricacies involved in leveraging this emerging technology to transform stakeholder and fan experiences at major sporting events. Finally, we outline and explain the obstacles, challenges, opportunities, and perspectives of digital twin technology at an intersection with sports events from a customer-centric perspective. The use of digital twins potentially enables the creation of hyper-realistic virtual replicas of sports venues, providing immersive and personalized experiences for spectators. This technology allows event organizers to optimize resource allocation, streamline logistics, and improve operational efficiency.

## 1. Introduction

Today, organizing mega sports events is seen as one of the societal and economic challenges (1–3) as well as rather controversial due to the complexity of satisfying diverse stakeholder expectations and mitigating negative impacts on host communities (3, 4). Consequently, mega sport events stakeholders are increasingly looking to advanced technologies like digital twins for solutions. For instance, the Paris 2024 Olympic Games plans to utilize a digital twin—an interactive virtual replica of the event. This technology can reduce the need for physical presence and travel, lowering carbon emissions while also providing new avenues for fan engagement. Virtual events accessed through digital twins allow remote fans to immerse themselves in compelling digital environments and interact with other attendees from around the world. As this example shows, digital twins enable stakeholders to negotiate the uncertainty and difficulties inherent in organizing massive events while promoting sustainability and innovating the fan experience (5). By harnessing emerging tech, stakeholders can work to unlock the marketing and community benefits of mega sports events while minimizing negative impacts and complexity. The turn towards innovative technologies demonstrates the focus on driving progress through digitally-powered solutions to realize the potential of major sporting events.

Technically, the real world can be reflected and re-created digitally. Semeraro et al. (6) argue that digital twin (DT) reflects the real-time operations of the physical system. In the context of events, A digital venue twin can be defined as a virtual interactive representation of an entire venue system. It is supposed to be hyper-realistic and uses real-time data and simulations for a user and user experience (UX). “Hyper-realistic” in the context of a digital twin, such as a digital venue twin, refers to the level of fidelity or realism achieved in the virtual representation of a physical system. Thus, the digital twin is designed to be so incredibly lifelike and detailed that it closely mirrors the real-world system it represents, often to the point where it can be challenging to distinguish between the virtual and physical environments.

Potentially, events' digital twins bring numerous benefits for all stakeholders (but not limited to): (1) lower maintenance costs, (2) reduced health, safety, and environmental risks, and (3) enhanced strategy to improve system performance, control, and maintenance, among others. For instance, SoFi Stadium in Los Angeles is set to utilize a digital twin to support daily management of the 3.1 million square foot, 70,000 seat venue and enhance the fan experience (7, 8). The virtual model contains information on equipment, conditions, and more to provide a holistic view of optimal stadium utilization. The virtual model of the stadium will allow for personalized wayfinding so fans can easily navigate to their seats. It also gives management data-driven insights to reduce concession stand wait times, optimize traffic flow, and make other improvements to benefit fans. On game days, SoFi Stadium's digital twin will assist new staff members or people unfamiliar with the massive arena by showing them exactly where they need to be (8). For major events like the Super Bowl that will draw huge crowds, this virtual map will be instrumental in creating a seamless experience for attendees.

To this end, digital twin software technology is capable to create an accurate virtual replica of a physical sporting event as a holistic system to boost productivity and streamline operations, making the event more inclusive, and accessible, both online and offline. Besides the organizers and local residents, spectators are another key event stakeholder (4, 9). While there is extensive discussion on the benefits of athletic training, stadium design, and sustainability in the context of digital twins, less focus has been placed on the customer-centric perspective. The customer-centric perspective is a well-adopted approach in business studies, with research examining event attributes that affect spectators' experience and satisfaction (10, 11).

The purpose is to adopt a customer-centric perspective the and introduce digital twin technology as a solution for mega-sport event management. This article focuses on the potentially drastic role of digital twin technology in modern sports events, explaining in detail different aspects of its impact. It contributes to understanding how and why sports venue digital twins make events more customer-centred by enhancing fan experiences and engagement. Subsequently, it aims to position digital twin technology as an innovative solution for mega-sport event management across various customer experience touchpoints. By examining the intersection of digital twins and sports events from a customer-centric lens, this article will elucidate the intricacies involved in leveraging this emerging technology to transform stakeholder and fan experiences at major sporting events. Finally, we outline and explain the obstacles, challenges, opportunities, and perspectives of digital twin technology at an intersection with sports events from a customer-centric perspective (12).

The main research question is “How (and why) do sports venue digital twin emerging technologies prospectively impact the sports spectators’ customer experiences?”

Furthermore, it involves a few auxiliary questions:

1. How may we describe the historical and intellectual development of the topic field?
2. What are the major trends and issues in the digital twin technologies market?
3. Who are the stakeholders and what is their interest and involvement with the digital twin technologies' massive dissemination, notably, in sports event management?
4. What are the opportunities and challenges with these trends and issues?
5. What are the catalysts of sports venue digital twin technologies in environmental issues (technology/ spectating /consumer expectations)?

To this end, we (1) understand and describe the nature, trajectory, and roles of technologies in sports events; (2) outline developing issues and trends that affect the present and future technologies deployment in sports entertainment through the perspective of spectators; (3) appreciate factors that contribute to transforming and enhancing customer-centric perspective in sports mega-events management; (4) recognize the importance of digital twin technologies' wide dissemination and their effect on event management; (5) understand, explain and describe how sports events are transforming through technology; (6) identify, disclose and introduce the role of the digital twins in future sports events from spectators perspective angle; (7) foresee the future research and development paths and directions. As a conceptual model, this article describes an entity of sports venues' digital twins' technology as an object and identifies related constructs and processes (13).

## 2. Theoretical prisms and perspectives

Melnick (14) suggests that various social forces, such as urbanization, individualism, interpersonal competition, technology, and geographical mobility, have led to an increase in the presence of strangers in people's lives. This, in turn, has made it more difficult to form close social ties with relatives, friends, neighbors, and colleagues. Consequently, individuals seek to fulfill their sociability needs through alternative means that are less personal, intimate, and private. Accordingly, Melnick (14) proposes that sports spectating has emerged as a significant urban structure where people come together not only for entertainment but also to enhance their social and psychological lives through quasi-intimate relationships that are available within this context. Sports spectating offers individuals an opportunity to engage in social interactions, albeit in a less personal and intimate manner (15, 16).

The changing nature of sociability experiences in sports spectating presents challenges and opportunities for sports managers. To maximize the potential of sports spectating facilities as a “gemeinschaft” (a community-oriented social structure), Melnick recommends that sports managers pay attention to the individual and communal aspects of their events. By doing so, they can increase spectator attendance while providing an important public service and spectators' infrastructure. This theory highlights the impact of social forces on people's social ties and suggests that sports spectating can serve as a platform for individuals to satisfy their sociability needs. By focusing on the communal and individual aspects of sports events, sports managers can create an environment, including infrastructure and technological equipment that promotes social interactions and enhances the overall spectator experience (5). Digital twin technology of a sporting venue can be seen as a digital infrastructure and technological equipment for sports spectating, including tools for fans' socializing and communications.

The next theoretical perspective has been brought by Glebova et al. (17) and described as a relocation of sports spectators' customer experiences (SSCX) as a theoretical prism on sport fans trough a customer-centric perspective in the framework of services management. Sports spectatorship is a popular pastime for many people, and in addition to attending live events, fans have various options for consuming sports content through different media platforms (12). These options include fan zones, broadcasts, podcasts, and mobile apps, which allow fans to stay updated on sports news, teams, athletes, events, organizations, and brands (18, 19). Television and radio broadcasts enable fans to watch or listen to live events when they cannot attend them in person. With the widespread availability of smartphones, tablets, internet access, mobile apps, and immersive technologies, sports fans now have the opportunity to have a 24/7 fan experience from anywhere.

Television networks and cable channels offer a wide range of sports programming, providing fans with numerous options for watching their favorite sports at both professional and other levels. The rapid development and diffusion of new technologies have opened up new opportunities for delivering Sports Spectating Customer Experiences (SSCX) and have transformed the forms and locations of these experiences. However, these changes are happening quickly and require further research to understand their impact. Glebova et al. (17) investigate the different locations of SSCX in the current sports fandom environment and they highlight the “physical” and “virtual” locations of SSCX. Physical location refers to the geographical place where a spectator is situated during the spectating experience, while virtual location refers to the way sports content is accessed. For instance, sports venues are tourism destinations (20). In today's digital world, the physical location of the fan does not matter in terms of content consumption; what matters is their virtual location. The study emphasizes that fans are “place-shifting” rather than “time-shifting,” as they access sports content from different locations using various devices and platforms. The concept of a “global stadium” (17) describes the aggregate of different types of sports fandom in the current internationalized and digitalized sports landscape. Glebova et al. (17) argue that with the increasing accessibility of digital sports media, fans are no longer confined to physical stadiums but can participate in the global stadium through social networks, online media, and other platforms. Some factors may influence the physical and virtual relocations of SSCX, including information accessibility, increasing mobility and flexibility of fans, the use of new tools for immersive and personalized experiences, and the changing habits of sports spectators.

Following the digital twins in manufacturing and engineering fields (21) and according to the Siemens Glossary, at large, there are three types of digital twins, namely (1) Product, (2) Production, and (3) Performance. The combination and integration of these three types as they evolve together are known as the unified digital “Thread” (22) and it can be seen as a holistic technological process with the perspective to be integrated into event management and spectating experience.

We can assume that with the dissemination and development of digital twin technology the culture of sports spectatorship and fans' habits will be undergoing significant changes due to the emergence of new forms of sports consumption and the availability of sports content through various channels (23). These changes are leading to relocations of SSCX, both in physical and virtual terms and a combination of factors such as information accessibility, fan mobility, immersive experiences, and evolving fan habits contribute to these relocations. The concept of a global stadium is proposed to encompass the physical and virtual locations of SSCX (17), but its gains new shadows in emergence with digital twins and a digital reflection of the real world in real-time.

## 3. Digital twin technology in sport spectacle

**Figure 1:**
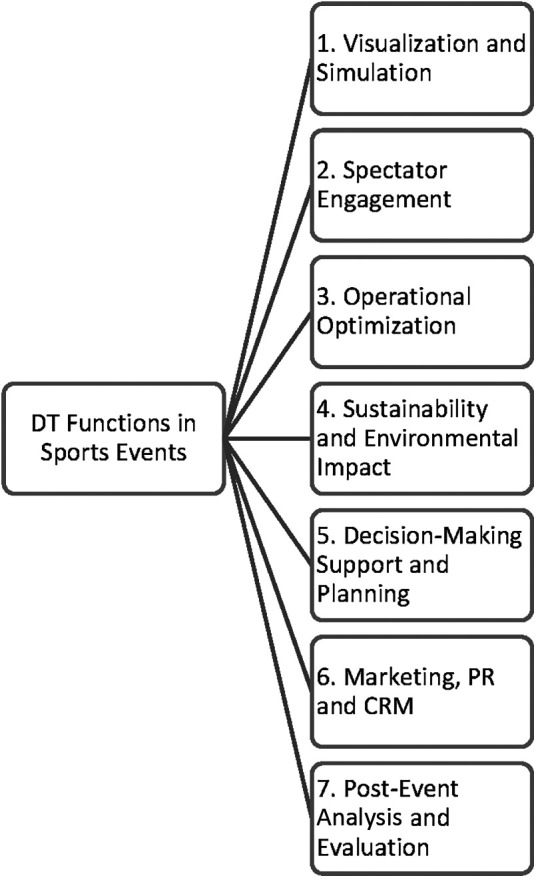
Digital twins functions in sports events.

Digital twin technology in sports events serves several functions that contribute to enhancing the overall experience for the stakeholders involved. There are some key functions of digital twin technology in sports events (Figure 1):

### 3.1. Visualization and simulation

Digital twins provide a virtual and interactive representation of the entire sports venue system, allowing stakeholders to visualize and simulate different aspects of the event (24). This includes simulating crowd flow, seating arrangements, lighting conditions, and other variables that impact the spectator experience (17).

### 3.2. Spectator engagement

Digital twins enable enhanced spectator engagement by offering immersive and interactive experiences (25). Spectators can explore virtual replicas of the venue, access real-time information (17), and interact with various elements of the event (23). This function fosters a more personalized and engaging experience for spectators (12).

### 3.3. Operational optimization

Digital twins assist in optimizing venue operations by leveraging real-time data and simulations. They can provide insights into crowd management (23), facility maintenance, resource allocation (26), and logistical planning (27). By optimizing these aspects, event organizers can improve operational efficiency and streamline processes (28).

### 3.4. Sustainability and environmental impact

Digital twins contribute to sustainability efforts in sports events by reducing environmental impact (29). By creating virtual experiences, digital twins can reduce the need for physical presence and associated carbon emissions (30). This function aligns with the growing demand for environmentally friendly practices (31, 32) in the sports industry (33).

### 3.5. Decision-making support and planning

Digital twins offer decision-making support tools for event organizers. By providing real-time data and analytics, they help inform decision-making processes (28), such as ticket pricing, security planning, and resource allocation (27). Digital twins enable event organizers to make data-driven decisions that improve planning and execution (12).

### 3.6. Marketing, customer and public relations, and sponsorship opportunities

Digital twins create new marketing and sponsorship opportunities by providing platforms for branding and advertising integration. Sponsors can leverage the digital twin experience to promote their products and engage with spectators in innovative ways (28). This function enhances revenue streams and strengthens sponsor-event and brand-consumer relationships, involving targeting and personalization (12).

### 3.7. Post-event analysis and evaluation

Digital twins capture vast amounts of data during the event, which can be analyzed post-event (34). This function allows event organizers to evaluate the success of the event, identify areas for improvement, and gain insights for future event planning and management, including operational needs and ecological event impact (32).

All these functions collectively contribute to improving the overall spectator experience, operational efficiency, and environmental and sustainability practices in sports events. Digital twin technology enhances various aspects of event management and opens up new possibilities for stakeholder engagement and innovation.

## 4. Digital twins functionality in the framework of sport event management

Digital twin technology in sports spectating serves a range of customer-centric functions that greatly enhance the spectator experience. Through the creation of virtual replicas of sports venues, digital twins offer an immersive viewing experience. Spectators can access these replicas using virtual reality (VR) or augmented reality (AR) technologies, providing them with a sense of being physically present at the event.

One of the key benefits of digital twins is the ability to personalize and customize the spectating experience. Spectators can select their preferred camera angles, access real-time statistics and information, and tailor their viewing preferences to match their interests. This level of personalization ensures that spectators have a tailored experience that meets their specific needs and preferences. Digital twins also promote interactivity and engagement (35). Spectators can actively participate in the virtual environment by interacting with features such as interactive screens, virtual fan zones, and social media integrations. This fosters a sense of community (as a whole) and engagement among spectators, making the experience more interactive and social.

Real-time information access is another advantage provided by digital twins. Spectators can stay up to date with live scores, player statistics, and event updates through digital interfaces, mobile applications, or connected devices. This real-time information keeps spectators informed and connected, enhancing their overall experience. One of the significant benefits of digital twins is remote accessibility (17). Spectators can engage in sports events from anywhere in the world, overcoming geographical constraints (36). This enables individuals who are unable to physically attend the event to still be part of the spectating experience, broadening the event's reach and inclusivity.

Digital twins also facilitate social interaction and community building among spectators. Virtual platforms and social media integrations enable spectators to connect with fellow fans, share their experiences, and engage in discussions at all stages of event management and marketing (34). The sense of belonging and community enhances the customer-centric environment, making the spectating experience more enjoyable. Furthermore, digital twins offer opportunities for feedback and surveys. Spectators can provide valuable insights and opinions to event organizers, allowing for continuous improvement and enhancement of future events. This gives spectators a voice and allows them to actively contribute to the shaping of the event.

Digital twin technology revolutionizes sports spectating by enhancing personalized, immersive, and interactive experiences (12, 17). By leveraging these customer-centric functions, event organizers can create a highly engaging and tailored environment that caters to the preferences and engagement of the spectators.

The implementation of digital twin technology in the Tour de France and Tour de France Femmes avec Zwift has revolutionized fan engagement and race operations. According to the NTT (37), this hyper-distributed environment, spanning remote regions of France, poses unique challenges in keeping fans engaged and informed across the world. Through data capture from cyclists, race vehicles, and race venues, a digital twin of the entire event is constructed, forming the foundation for the world's largest connected stadium. Real-time data analytics and AI enable the delivery of enhanced, data-driven storytelling to fans, improving their experience with new visualizations and narratives each year. Additionally, the technology facilitates faster, data-driven decision-making for race operations, integrating information that was previously isolated. The introduction of an AI-powered digital human avatar named “Marianne” further enhances interactivity and information dissemination, promising to enrich the overall fan experience. The continuous evolution and advancement of digital twin technology ensure its pivotal role in shaping the future of the Tour de France and similar events.

## 5. Guidelines of DT development and implementation

The historical and intellectual development of digital twin technology in sports event management can be traced back to the convergence of various fields, including virtual reality, simulation, and data analytics (38). The concept of digital twins originated in the field of engineering, where virtual replicas of physical assets were used for design optimization, predictive maintenance, and performance monitoring (27, 28). This concept has since expanded to other industries, including sports.

In the context of sports events, the development of digital twin technology has been driven by advancements in computing power, data analytics, and the wide dissemination of technologies in the sport spectacle field (12, 36). Initially, digital twins were used to model and simulate specific aspects of sports venues, such as crowd flow or lighting conditions (25). However, with the increasing availability of real-time data from IoT devices and the advancement of virtual reality technologies, digital twins now aim to create holistic and hyper-realistic representations of entire venue systems.

The intellectual development of the field involves the exploration of how digital twin technology can be applied to sports event management. Researchers and practitioners have investigated the potential benefits of digital twins in optimizing operations (26, 27), enhancing spectator experiences (12), and improving sustainability in sports events (30).

The digital twin technologies market is witnessing several major trends. First, there is a focus on integrating advanced data analytics and machine learning techniques to extract meaningful insights from the vast amount of real-time data collected by IoT devices in sports venues (37). This enables better decision-making, predictive modeling, and optimization of various aspects, such as crowd management (important for security and control), facility maintenance, and resource allocation (26). Second, there is a growing trend toward immersive experiences through virtual reality and augmented reality technologies. Digital twins allow spectators to virtually explore and interact with sports venues, creating a more engaging and personalized experience. This trend is driven by the increasing demand for unique and interactive fan experiences. Third, there is an emphasis on the integration of digital twin technology with broader event management systems. This includes integrating with ticketing platforms, broadcasting systems, and analytics tools to provide a holistic and interconnected approach to sports event management.

One of the key challenges seems to be ensuring robust data privacy and security measures. As digital twins rely on real-time data collection from various sensors and devices, it is crucial to protect sensitive information and prevent unauthorized access. Furthermore, the complexity of capturing and simulating the dynamic and multidimensional nature of sports events poses a significant technical challenge, including user experience and data management, and visualization.

Various stakeholders play a role in the dissemination of digital twin technologies in sport event management. These stakeholders include sports event organizers, venue operators, technology providers, sponsors, broadcasters, and spectators. Sports event organizers and venue operators are interested in leveraging digital twin technology to enhance operational efficiency, optimize resource allocation, and improve the overall event experience for spectators. They are motivated by the potential to streamline logistics, reduce costs, and create unique and immersive experiences that attract and retain fans. Technology providers develop and offer digital twin solutions tailored to the sports industry. Their involvement includes designing and implementing digital twin platforms, integrating IoT devices, and providing analytics capabilities to extract insights from the data collected by the digital twins. Their interest lies in offering innovative solutions that address the specific needs of sports event management. Sponsors and broadcasters have a vested interest in digital twin technologies as they provide opportunities for enhanced branding, advertising, and content distribution. Digital twins can offer new avenues for sponsor integration and engagement with spectators, while broadcasters can leverage immersive experiences to provide more engaging and tailored content to viewers. Potentially, an event and venue digital replica has a drastic experiential potential in an immersive environment, including all forms of extended reality. Spectators, the end-users of digital twin experiences, are increasingly interested in personalized and interactive fan experiences. They expect digital twin technologies to provide a more immersive and engaging way to connect with the sports event, access information, and participate in the event remotely.

The trends and issues in digital twin technologies for sports events present both opportunities and challenges. Digital twins enable event organizers to simulate and optimize various aspects of the event, such as seating arrangements, crowd flow, and facility layouts, leading to better event planning and design. Immersive experiences offered by digital twins can create more engaging and personalized interactions for spectators, leading to increased fan satisfaction and loyalty (12). By leveraging real-time data and analytics, digital twins can help optimize resource allocation, maintenance schedules, and facility operations (26), leading to increased efficiency and cost savings. The insights derived from digital twins can inform marketing and business strategies, helping event organizers make data-driven decisions to attract sponsors, improve revenue streams, and enhance the overall fan experience (23). At the same time, on the other hand, integrating complex technologies (12), such as digital twins can be challenging due to compatibility issues, data synchronization, technology acceptance (31), and system interoperability. Collecting and analysing real-time data from IoT devices raises concerns about data privacy and security (12). Safeguarding sensitive information and ensuring compliance with data protection regulations are critical challenges. Furthermore, implementing digital twin technologies can require significant resources and investments in infrastructure, technology platforms, and sophistically skilled personnel with particular technical experience. Scaling the technology to large-scale sports events can be a challenge due to computational and logistical complexities. Also, creating accurate and reliable virtual replicas of sports events is a complex task (25). Probably, ensuring that the digital twin simulations accurately represent real-world dynamics and can be validated against actual event data poses a challenge.

Sports venue digital twin technologies have the potential to address environmental issues in several ways (30). For instance, the convergence of technologies such as IoT, data analytics, and virtual reality enables more efficient resource management and reduces energy consumption in sports venues (25). For example, digital twins can optimize lighting, HVAC systems, and crowd flow to minimize energy usage and environmental impact (32).

Modern consumers (spectators) increasingly value sustainability and expect sports events to align with their environmental consciousness (18). Digital twin technologies can facilitate remote participation and reduce the need for physical travel to events, thereby reducing carbon emissions associated with spectator mobility. Consumers, including sports fans, are becoming more conscious of environmental issues and prefer brands and events that demonstrate environmental responsibility. Sports venues utilizing digital twin technologies to improve sustainability can attract environmentally conscious consumers. Adopting digital twin technologies in sports event management showcases innovation and leadership in sustainability practices (32). By implementing advanced technologies to create more environmentally friendly events, sports organizations can set industry standards and inspire others to follow suit.

These catalysts collectively drive the adoption and implementation of sports venue digital twin technologies to address environmental concerns, improve sustainability practices (39), and align with the expectations of environmentally conscious spectators and consumers.

Obviously, the field of digital twin technology in sports event management is still evolving, and further research and practical applications are necessary to fully understand and capitalize on its potential impact and benefits.

### 5.1. Four Ps of digital twins and digital thread

**Figure 2:**
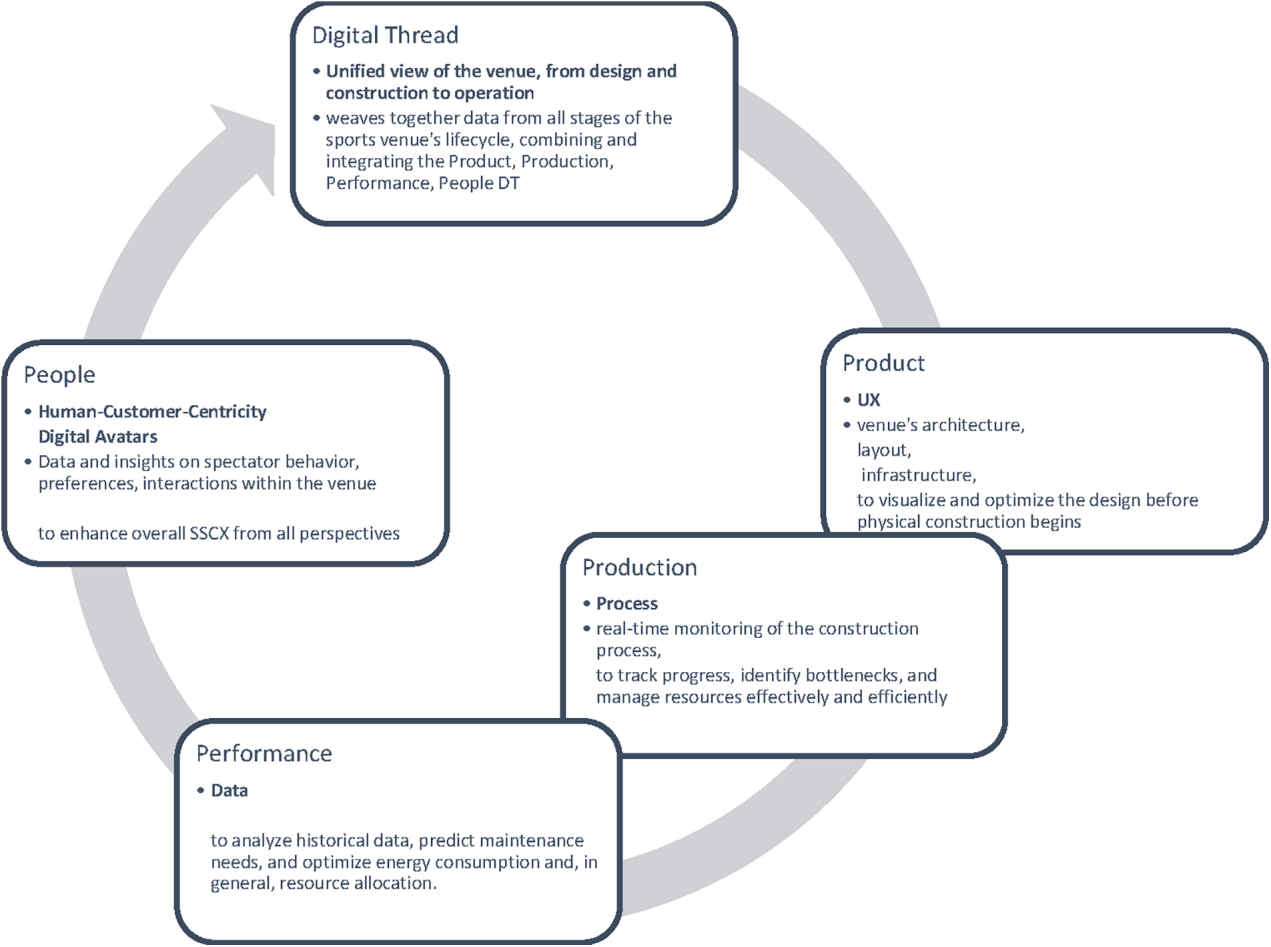
Digital thread and four Ps of sport event digital twins.

Applying the concept of a digital twin to a sports venue involves leveraging its potential applications based on the three types of digital twins: product, production, and performance (21, 22, 40) in sport event management from a spectator-centric perspective but taking into account a holistic stakeholder approach (Figure 2).

#### 5.1.1. Product digital twin

The Product Digital Twin represents the virtual replica of the sports venue during its design and planning stages. It captures detailed information about the venue's architecture, layout, and infrastructure, enabling architects, engineers, and stakeholders to visualize and optimize the design before physical construction begins. By simulating various scenarios, potential issues and inefficiencies can be identified and addressed, leading to cost savings and enhanced functionality. Throughout the lifecycle of the sports venue, the Product Digital Twin serves as a reference for maintenance, refurbishment, and renovation activities, ensuring that changes and updates are aligned with the original design intent.

#### 5.1.2. Production digital twin

The Production Digital Twin focuses on the construction phase of the sports venue. It facilitates real-time monitoring of the construction process, enabling project managers to track progress, identify bottlenecks, and manage resources effectively and efficiently. By integrating data from construction equipment, materials, and workers, the Production Digital Twin provides a holistic view of the construction site, allowing for proactive decision-making and timely interventions. Any deviations from the original design can be quickly detected and rectified, reducing delays and cost overruns. Furthermore, the Production Digital Twin aids in ensuring compliance with safety and quality standards during the construction phase.

#### 5.1.3. Performance digital twin

Once the sports venue is operational, the Performance Digital Twin comes into play. It continuously gathers data from various sensors and IoT devices installed throughout the venue, capturing real-time information about crowd movement, facility usage, environmental conditions, and equipment performance. This data is used to optimize venue operations, enhance spectator experiences, and improve overall safety and security. The Performance Digital Twin allows venue operators to analyze historical data, predict maintenance needs, and optimize energy consumption and, in general, resource allocation. Additionally, it assists in delivering personalized experiences to fans, enhancing their engagement and satisfaction.

#### 5.1.4. People digital twin: extending the typology and the digital thread

We believe that adding a “People Digital Twin” as the fourth “P” to the DT typology is a valuable extension, especially in the context of a customer-centric approach. The People Digital Twin focuses on capturing and analyzing data related to the spectators and other stakeholders within the sports venue. It gathers data on spectator behavior, preferences, and interactions within the venue. This may include tracking movements, identifying crowd hotspots, monitoring concession stand usage, and analyzing seating preferences. The People Digital Twin is instrumental in enhancing the overall spectator experience. It allows venue operators to gain a deeper understanding of spectator needs and preferences, enabling them to make data-driven decisions to improve services, optimize seating arrangements, and tailor offerings. It can also aid in crowd management, helping to ensure safety and security during events.

Digital twins embrace both spaces and their digital representations, as well as real people and their digital avatars. A digital avatar is defined as a kind of visual presentation of an attendee or visitor (41).

#### 5.1.5. Integration of “people” with the three existing Ps

The People Digital Twin is closely integrated with the existing Product, Production, and Performance Digital Twins through the Digital Thread. Data collected from the People Digital Twin can be shared and analyzed in conjunction with data from the other digital twins. This integration allows for a holistic view of the venue that includes both physical and human elements. Insights gained from the People Digital Twin can inform decisions related to design, construction, and operations, ultimately leading to a more customer-centric and satisfying experience.

The inclusion of the People Digital Twin completes a comprehensive approach to sports venue management, where the needs and experiences of spectators and stakeholders are central. Placing “people” as a priority ensures that the sports venue is designed, built, and operated with the aim of delivering exceptional experiences to spectators and other stakeholders.

The four “P”s, when integrated through the Digital Thread, provide a wealth of data that empowers stakeholders to make informed decisions, optimize resources, and continuously improve the venue's design, construction, and operations. In general, incorporating the People Digital Twin into the theoretical model enriches the customer-centric approach, making it more robust and responsive to the needs and preferences of the people who attend and engage with the sports venue. This extension enhances the model's ability to create a truly immersive and satisfying experience for all stakeholders.

#### 5.1.6. Integration of all Ps through the digital thread

The Digital Thread weaves together data from all stages of the sports venue's lifecycle, combining and integrating the Product, Production, Performance Digital Twins (40), and, as we suggest the People Digital Twin as well. This comprehensive approach allows stakeholders to have a unified view of the venue, from its design and construction to its operational phase. As the digital twins evolve together, insights gained from one stage can inform and optimize subsequent steps and settings, promoting continuous improvement and efficiency throughout the venue's lifecycle (33) and event management (42).

The application of a sports venue digital twin offers tremendous benefits by enhancing design, construction, and operational processes. The combination of Product, Production, Performance, and People Digital Twins through the Digital Thread empowers stakeholders with valuable insights and data-driven decision-making, ultimately resulting in a more efficient, sustainable, and engaging sports venue (33).

This approach provides stakeholders with a unified view of the venue, from design and construction to operation. It allows insights gained from one stage to inform and optimize subsequent steps, promoting continuous improvement and efficiency. Integrating the three digital twins through the Digital Thread empowers stakeholders with valuable insights and data-driven decision-making, leading to a more *efficient, sustainable, and engaging* sports venue.

## 6. Explaining digital twins in sport event management

### 6.1. Three pillars principles of integration, dissemination, and maintenance of DT

**Figure 3:**
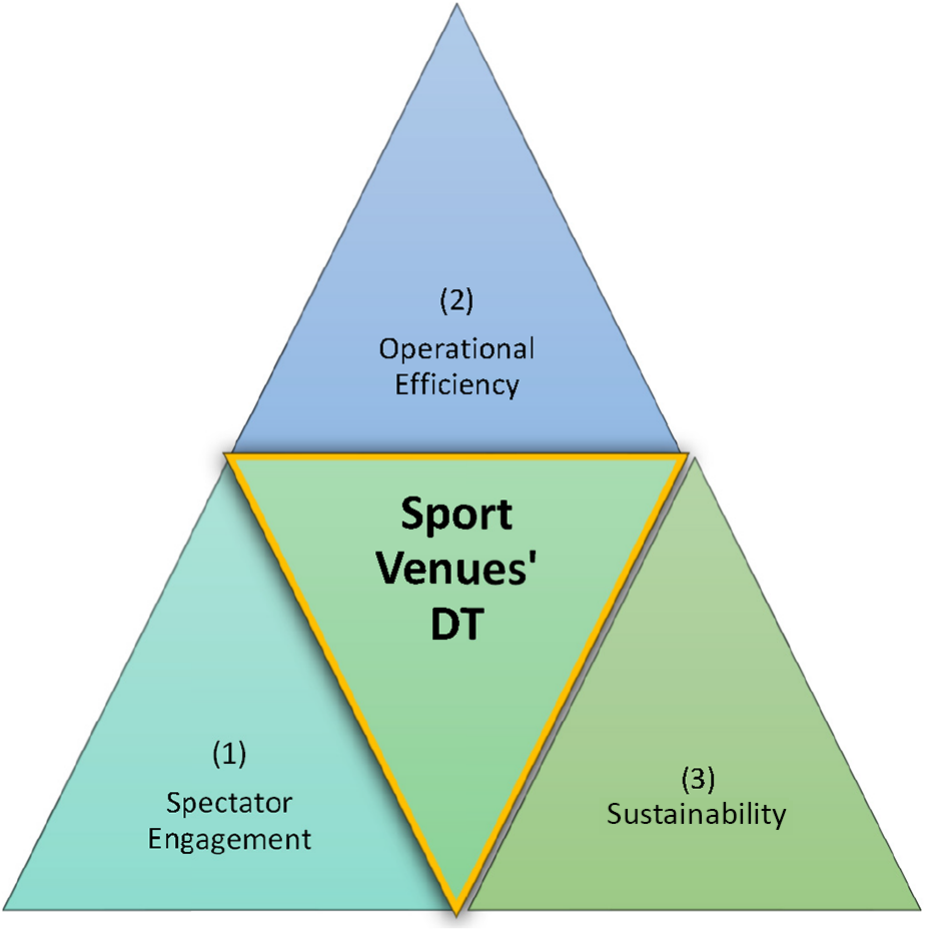
Sport event digital reflection: three pillars of digital twins.

According to Jaakola (13), explaining the evolving phenomenon involves related theoretical constructs and a reliable framework. Based on the information provided, we propose a framework, implying that the integration of digital twin technology in sports event management has the potential to create customer-centric experiences and enhance various aspects of sports events, including three main pillars referring to principles of integration, dissemination, and maintenance of DT: (1) spectator engagement, (2) operational efficiency, and (3) sustainability (Figure 3).

Subsequently, we can propose that by leveraging digital twin technology, sports event organizers can create hyper-realistic virtual replicas of sports venues, enabling immersive and personalized experiences for spectators. This, in turn, enhances fan engagement, satisfaction, and loyalty (25). Furthermore, it lets us suggest that digital twin technology facilitates optimized venue operations by utilizing real-time data and simulations. Event organizers can leverage this technology to improve resource allocation, optimize facility layouts, and streamline logistics, leading to increased operational efficiency and cost savings (23, 26, 39). Also, it highlights the potential of digital twin technology in addressing environmental issues in sports events (33). Among other dimensions of sustainability (39), by reducing the need for physical attendance and minimizing associated carbon emissions, digital twins may contribute to enhanced environmental sustainability in the sports industry (32), fostering a meaningful consumption philosophy. Thus, the “Event Digital Reflection” (Figure 3) emphasizes the transformative potential of digital twin technology in reshaping the way sports events are organized, maintained, and experienced. It underscores the importance of integrating customer-centric approaches, advanced data analytics, and immersive technologies to create personalized, compelling, and sustainable sports event experiences.

### 6.2. Entry barriers in the digital twin sports arena

Gaining access to a Digital Twin sports event is not as straightforward as simply purchasing a ticket to a traditional game. Fans would need to purchase VR headsets, motion controllers, subscription fees to platforms, and a designated space for movement. These prerequisites add barriers to fans who are not familiar with the digital twins thus creating a divide, even enthusiasts would feel overwhelmed by switching between various virtual spaces where they suffer from cognitive overload. Additionally, those with limited living spaces may find it challenging to meet the spatial demands of a comprehensive Digital Twin experience.

### 6.3. Data privacy digital twin arena

Hosting and Participating in a Digital Twin sports event involves sharing a significant amount of data, some of which may be sensitive or personal. Given the lack of regulation and legality in privacy control and data management in Digital Twin sports environments. At present, the guidelines for data management are still evolving, leaving a set of urgent questions that require attention.

In this context, the concept of consumer-centric organizational learning becomes particularly relevant. Sports organizations must engage in an ongoing, embodied practice of learning that is not only internal and external but also consumer-focused. It is important that the organizer constantly *y*, a variety of interactive phenomena arise from a direct and engaged participation in the embodied ambiguous life-world experience of the embodied consumer experience to provide a more accessible and safe space for the consumer. The notion of “inter-learning” is crucial here, as organizations need to adapt to these consumer-centric entry barriers, viewing them not merely as technological hurdles but as challenges requiring a consumer-focused approach for resolution.

## 7. Conclusion

The integration of digital twin technology in sports event management presents exciting opportunities for enhancing the spectator experience, optimizing venue operations, and promoting environmental sustainability. The use of digital twins potentially enables the creation of hyper-realistic virtual replicas of sports venues, providing immersive and personalized experiences for spectators. This technology allows event organizers to optimize resource allocation, streamline logistics, and improve operational efficiency.

Furthermore, digital twin technology offers the potential to address environmental concerns by reducing the need for physical attendance and minimizing associated carbon emissions. By leveraging real-time data and simulations, event organizers can make data-driven decisions that improve sustainability practices in sports events.

### 7.1. Limitations and research directions

In scrutinizing the transformative potential of digital twin technology within the management paradigm of mega sports events, it is imperative to underscore the conspicuous absence of robust empirical evidence and a comprehensive literature base on this nascent subject. The research endeavor, while commendable in its exploration of the purported benefits of digital twins, operates within the confines of a conspicuously sparse body of empirical evidence. This scarcity poses a dual challenge, as it not only accentuates the study's reliance on theoretical suppositions but also underscores the paucity of substantiated insights into the practical implications and challenges associated with the adoption of digital twin technology in the context of mega sports events.

Furthermore, the primary focus on extolling the theoretical merits of digital twins may inadvertently obscure the pressing need for empirical investigations, thus limiting the depth of scholarly understanding. The absence of empirical underpinnings may impede the nuanced comprehension of the actual dynamics, challenges, and efficacy of digital twin implementation within the unique milieu of mega sports events.

This notable gap in empirical evidence and scholarly discourse underscores the imperative for future research endeavors to bridge this knowledge lacuna. A concerted effort is required to bolster the empirical foundation, allowing for a more substantive and evidence-based elucidation of the implications, challenges, and effectiveness of digital twin technology in mega sports event management. The scholarly community is urged to contribute empirical studies that scrutinize the practical applications and outcomes of digital twin implementation in diverse mega sports event contexts, thereby enriching the current discourse and paving the way for a more robust understanding of the subject matter.

### 7.2. Theoretical and practical implications

However, it is important to acknowledge the challenges and complexities associated with implementing digital twin technology, such as data privacy and security, integration of diverse technologies, and ensuring the accuracy and reliability of virtual representations. Overcoming these challenges will require further research, technological advancements, and collaboration among stakeholders.

The adoption development and dissemination of digital twin technology in sports event management holds great promise for creating customer-centric experiences (SSCX), optimizing operations, and advancing sustainability. Continued exploration and implementation of this technology can lead to innovative and transformative changes in the way sports events are organized, experienced, and managed.

This technological milieu, furthermore, holds promise in mitigating environmental concerns by attenuating the imperative for physical attendance, thereby minimizing associated carbon emissions through the judicious application of real-time data and simulations. Nevertheless, the assimilation of digital twin technology is not bereft of formidable challenges, prominently encompassing concerns of data privacy, security imperatives, seamless integration of diverse technological frameworks, and the assurance of veracity in virtual representations. A requisite acknowledgment of these challenges is imperative, concomitant with an assiduous commitment to further research endeavors, technological advancements, and collaborative endeavors among pertinent stakeholders.

Theoretical and practical implications emanate from this technological integration. User-centric interfaces for virtual replicas necessitate careful design considerations, as do systems for personalized all types of contents delivery. Equally pivotal is the instigation of comprehensive training programs for venue personnel, ensuring adept utilization of digital twin data. Concurrently, investment in artificial intelligence algorithms, deployment of Internet of Things devices for real-time monitoring, and fortification of cybersecurity measures are indispensable facets of practical implementation (43).

The standardization of industry protocols, fostering collaborative frameworks among technology providers, and a stringent commitment to the accuracy and reliability of digital twin representations are pivotal endeavors. Beyond these, stakeholder collaboration, innovation in user experiences, and sustained research and development initiatives emerge as fundamental contributors to the transformative potential of digital twin technology within the realm of sports event management. These measures collectively engender a theoretical and practical landscape ripe for innovative and sustainable transformations in the orchestration, experience, and administration of sports events.
